# Comparative study of prophylaxis with high and low doses of voriconazole in children with malignancy

**DOI:** 10.18502/cmm.6.4.5331

**Published:** 2020-12

**Authors:** Sviatlana L. Kandaurava, Kseniya S. Baslyk, Alexandr A. Migas, Anna V. Hill, Oleg I. Bydanov, Volha A. Mishkova, Olga V. Aleinikova

**Affiliations:** 1 Infection Control Department, Belarusian Research Center for Pediatric Oncology, Hematology, and Immunology, Minsk, Belarus; 2 Laboratory of Genetic Biotechnology, Scientific Department, Belarusian Research Center for Pediatric Oncology, Hematology, and Immunology, Minsk, Belarus; 3 Group of Molecular Biology and Transplant Processing, Belarusian Research Center for Pediatric Oncology, Hematology, and Immunology, Minsk, Belarus; 4 Automated Control Systems Department, Belarusian Research Center for Pediatric Oncology, Hematology, and Immunology, Minsk, Belarus; 5 Laboratory of Molecular and Genetic Research, Scientific Department, Belarusian Research Center for Pediatric Oncology, Hematology, and Immunology, Minsk, Belarus; 6 Laboratory of Cellular Biotechnology and Cytotherapy, Scientific Department, Belarusian Research Center for Pediatric Oncology, Hematology, and Immunology, Minsk, Belarus

**Keywords:** Fungal infections, Children, Prophylaxis, Voriconazole

## Abstract

**Background and Purpose:**

Children with acute myeloid leukemia and relapses of leukemia are at high risk of developing fungal infections and need antifungal prophylaxis. This study aimed to compare the efficacy and toxicity of two different dosage regimens of voriconazole (VRC) during prophylactic administration in children with malignancy and neutropenia.

**Materials and Methods:**

This prospective study was conducted at the Belarusian Research Center for Pediatric Oncology, Hematology, and Immunology from May 2017 to December 2019. The present study included 21 Caucasian patients with malignant hematological diseases (20 patients with acute myeloid leukemia and relapses of leukemia and 1 patient with Non-Hodgkin's lymphoma) aged 2-18 years. All patients were randomly divided into two groups that received different dosage regimens of VRCZ prophylaxis. Patients in the “high-dose” group received VRCZ at a dose of 9 mg/kg twice a day PO, or 8 mg/kg twice a day IV without a loading dose (children of 2-11 and adolescents and of 12-14 years old with <50 kg weight body), or a dose of 4 mg/kg twice a day PO or IV (adolescents of 12-14 years old with ≥50 kg body weight and all adolescents over 14 years old). Patients in the “low-dose” group received VRCZ at a dose of 4 mg/kg twice a day, PO or IV, without a loading dose (children of 2-11 and adolescents of 12-14 years old with <50 kg body weight), or at a dose of 3 mg/kg twice a day, PO or IV (adolescents of 12-14 years old with ≥ 50 kg body weight and all adolescents over 14 years old). When neutropenia recurred (after the next chemotherapy block), the patients were re-randomized and prophylaxis was resumed in the absence of fungal infection. Therefore, some patients (n=12, 57%) entered the study several times (maximum four times, after each chemotherapy block). In total, 21 patients experienced 40 episodes of VRCZ prophylaxis.

**Results:**

In the high-dose group (n=20 episodes of prophylaxis), invasive fungal infections (IFI) signs were recorded in one (5%) case. In the low-dose group (n=20 episodes), IFI signs were observed in six (30%) cases (*P*=0.0375). The residual serum concentration was significantly higher in patients who received high doses of VRCZ (*P*<0.0001). Most patients with IFI (n=6, 86%) had a mean value (i.e., <0.74 μg/ml) of the residual serum concentration of the medication. Median of the first signs of fungal infection was 22 days from the start of prophylaxis. The dosage was the only highly significant factor that affected the metabolism of VRCZ.

**Conclusion:**

The likelihood of IFI was significantly lower in children who prophylactically received VRCZ in high doses (*P*=0.0375) and had ≥ 0.74 μg/ml residual serum concentration of the medication (*P*=0.0258). Residual serum concentration of VRCZ reached a plateau by day sixth of the treatment. In children, the dosage was the only highly significant factor affecting the metabolism of VRCZ.

## Introduction

The ubiquitous distribution of fungi justifies their presence in the air, soil, water, food, environmental objects, and on human mucous membranes [ 1 ]. Many types of fungi can cause serious infections in immunocompromised individuals, especially those with cancer and hematological malignancies,.

Among molds, *Aspergillus* spp. remains the dominant causative agent of invasive fungal infections (IFI). Most often, invasive aspergillosis (IA) occurs in patients with acute myeloid leukemia (AML) and relapses of leukemia, lung transplant recipients, and patients with chronic obstructive pulmonary disease who receive corticosteroids [ 2 ]. 

Voriconazole (VRCZ) is the medication of choice for the treatment of IA [ 3 ]. It belongs to the azole class and has a wide spectrum of antifungal activity. The VRCZ is also effectively used for antifungal prophylaxis and has adverse effects, such as increased hepatic enzymes, phototoxic reactions, and ophthalmic disorders. The VRCZ is metabolized by cytochrome P450 Isoenzymes (*CYP2C19* to a greater extent, as well as *CYP2C9* and *CYP3A4*) which inhibit their activity. Inhibitors or inducers of these Isoenzymes can cause an increase or decrease in the concentration of VRCZ in plasma. In turn, VRCZ can increase plasma and intracellular concentrations of substances that are metabolized by these Isoenzymes; therefore, multiple medication interactions are characteristic of VRCZ [ 4 ]. 

The *CYP2C19* polymorphism also affects the metabolism of VRCZ. It is known that in different populations, there are distinct genetic controls of some isozymes of the cytochrome P450 system [ 5 ]. This phenomenon causes the unusual enzymatic activity of this system in different people. For example, the clearance of medications metabolized by *CYP2C19* varies from 5 to 20 times in different individuals and ethnic groups primarily due to the consequences of genetic polymorphism [ 6 , 7 ]. This phenomenon is also the result of non-genetic factors (e.g., internal medication interactions [ 7 ], age [ 8 ], pregnancy, and possibly other disorders [ 9 , 10 ]). Based on metabolic ability, all people can be classified as:

- Individuals with slow metabolism (SM)
- With intermediate metabolism (IM)
- With fast metabolism (FM)
- With superfast/ultrafast metabolism (UM)

The *CYP2C19* genotype is represented by the "wild" type (*1) and two mutant alleles *2 (*2) and *3 (*3). The most common alleles for *CYP2C19* slow metabolizers are *2 and *3 (*2⁄*2, *3⁄*3, or *2⁄*3). The *1 allele represents the genotype of fast metabolizers while intermediate metabolizers have *1⁄*2 or *1⁄*3 genotypes. There is also the *CYP2C19* genotype that has the *17 alleles which are associated with increased function of the ultrafast metabolizers [ 6 , 7 , 11 ]. There is evidence of the effect of food and a low concentration of azole levels in oral administration [ 12 - 14 ]. All of the above-mentioned factors necessitate routine monitoring of the residual serum concentration of VRCZ during its administration [ 15 ]. 

According to previous research, the target therapeutic level of residual serum concentration of VRCZ is 1-6 μg/ml [ 16 - 20 ]. Moreover, the target prophylactic level of residual serum concentration of VRCZ for adults is defined in the same range; however, currently, little research has focused on this indicator in children. Safety and effectiveness of the use of VRCZ have not been determined in children under two years of age. Therefore, further studies are needed to determine the optimal dosage regimen for the prophylactic use of VRCZ in children. 

## Materials and Methods

According to ECIL-8, patients with AML and relapsed acute lymphoblastic leukemia are at high risk of developing IFI ( > 10%). Furthermore, the frequency of IFI in pediatric patients with AML has been reported to be 10–27% [ 21 , 22 ]. 

 This study was approved by the Ethics Committee of Belarusian Research Center for Pediatric Oncology, Hematology and Immunology, Minsk, Belarus (Document No. 13 from December 28th, 2016). The present research was performed on 21 Caucasian patients with malignant hematological diseases (20 patients with acute myeloid leukemia (AML) and relapses of leukemia and 1 patient with Non-Hodgkin's lymphoma) aged 2-18 years. Acute leukemia was diagnosed according to the French–American–British classification and the treatment of the studied patients was administered based on the international protocols approved in our Center. The patients orally received VRCZ as antifungal prophylaxis for the period of chemotherapy induction (for patients with AML) and chemo-induced neutropenia. If oral administration was not possible, VRCZ was administered intravenously. 

Prophylaxis was carried out during the entire period of neutropenia (absolute neutrophil count <500/l) and ended with the restoration of granulocyte levels (ANC≥500/l) or the occurrence of a fungal disease. After each chemotherapy block, patients were randomly divided into two groups with different dosage regimens of VRCZ. When neutropenia recurred (after the next chemotherapy block), patients were re-randomized and prophylaxis was resumed (in the absence of fungal infection). Patients with signs of infection at the start of prophylaxis were excluded from the study. It should be noted that this study design was chosen due to the small number of patients. 

All neutropenia episodes were divided into two groups with different dosage regimens of VRCZ. In each of the two groups, there were 20 episodes of VRCZ prophylaxis during neutropenia. Patients in the high-dose group (n=20 episodes) received a high dose of VRCZ as prophylaxis, while patients in the low-dose group (n=20 episodes) received a low dose of it. 

The study power was 76.3% based on randomized group allocation of 20 episodes. Moreover, the expected frequency of fungal infection in the high-dose and low-dose prophylaxis groups were < 10% and -10-27%, respectively [ 22 ]. 

Patients in the high-dose group received VRCZ at a dose of 9 mg/kg twice a day PO or 8 mg/kg twice a day IV without a loading dose (children of 2-11 and adolescents of 12-14 years old with <50 kg body weight), or at a dose of 4 mg/kg twice a day, PO or IV (adolescents of 12-14 years old with ≥50 kg body weight and all adolescents over 14 years old). 

Patients in the low-dose group received VRCZ at a dose of 4 mg/kg twice a day, PO or IV, without a loading dose (children of 2-11 and adolescents of 12-14 years old with <50 kg body weight), or at a dose of 3 mg/kg twice a day, PO or IV (adolescents of 12-14 years old with ≥50 kg body weight and all adolescents over 14 years old). It should be noted that the bodyweight of the patients was measured on a daily basis. 

Prophylaxis was considered effective if, during its implementation, patients did not develop a probable or proven IFI (as defined by the European Organization for Research and Treatment of Cancer/Mycosis Study Group, EORTC/MSG 2019) [ 23 ]. If the signs of IFI appeared during medical prophylaxis, the latter was considered ineffective; therefore, it was stopped and the study of these patients was finished. The IFI was diagnosed based on the EORTC/MSG guideline, 2019 [ 23 ]. 

To study the factors that affect the metabolism of VRCZ, the residual serum concentration was measured. The concentration control was carried out daily during the first eight days of the prophylaxis period and after that, every 48 h until the medication was stopped. The residual serum concentration of VRCZ was determined by high-performance liquid chromatography-mass spectrometry (HPLC-MS). The VRCZ (manufactured in Sigma Aldrich, USA) and VRCZ-d3 (manufactured in Cerilliant, USA) were used as standards. 

All patients underwent the determination of *CYP2C19* gene polymorphisms which was carried out using the Sanger sequencing method. The chroma-tograms were analyzed using the Sequencing Analysis software (version 5.2) (Applied Biosystems, USA). Comparison of wild type nucleotide sequences obtained by sequencing was performed using the Ensembl genomic browser (http://www.ensembl.org). 

**Statistical analysis**

The nonparametric Mann-Whitney U test was used for the confirmation of the hypothesis of the presence of statistical differences between the two groups. Besides, a box and whisker diagram (box plot) was used to graphically present the quartile-median distribution of quantitative data. In addition, the criterion χ2 was used to compare the categorical data in the two groups. It should be mentioned that a p-value of less than 0.05 was considered statistically significant in all the statistical tests. Significance of the outcome classification was assessed using the receiver operating characteristic (ROC) analysis, and the rating was deemed to be significant at the area under the curve (AUC) of > 0.65. Based on the ROC analysis, we determined the outcome separation with maximum sensitivity and specificity. This separation was the cut-off point in the classification. Logistic regression was used for the multivariate analysis. For calculations, we used the R software (version 3.2.0), R-foundation for Statistical Computing, GNU GPL license.

## Results

This study was performed on 21 patients with malignant hematological diseases within the age range of 2-18 years old. In total, 15 (71%), 5 (24%), and 1 (5%) patients had AML, relapse of acute leukemia (four had a relapse of myeloid leukemia and one has a relapse of lymphoblastic leukemia), and Non-Hodgkin's lymphoma. Most patients (n=20, 95%) were at high risk of developing IFI while one of them was in the intermediate-risk group for the development of IFI (the patient with Non-Hodgkin's lymphoma). Some patients (n=12, 57%) entered the study several times (maximum four times, after each chemotherapy block). In total, 21 patients generated 40 episodes of antifungal prophylaxis with VRCZ, each of which was evaluated separately. All neutropenia episodes were divided into two groups with different dosage regimens of VRCZ. In each of the two groups, there were 20 episodes of VRCZ prophylaxis during neutropenia (Table 1). It should be mentioned that patients at a low risk of IFI were not included in the study. 

In the high-dose group, signs of a probable or proven IFI [ 23 ] were observed in 1 (5%) case (a proven case of disseminated fusariosis). Furthermore, in the low-dose group, symptoms of fungal infections were recorded in six (30%) cases (five cases of probable aspergillosis, one case of proven disseminated mucormycosis in lung, liver, and spleen damage) (*P*=0.0375). Signs of a probable or proven yeast infection were not observed in any of the groups (Table 1). 

**Table 1:** Number of high-dose/low-dose prophylaxis episodes for each patient

Patient	Number of high-dose/low-dose prophylaxis episodes	low-dose	high-dose
1	3	0	3
2	2	1	1
3	2	1	1
4	4	4	0
5	2	1	1
6	3	2	1
7	2	1	1
8	2	0	2
9	3	0	3
10	2	0	2
11	4	2	2
12	2	1	1
13	1	1	0
14	1	0	1
15	1	1	0
16	1	1	0
17	1	1	0
18	1	1	0
19	1	1	0
20	1	0	1
21	1	1	0

**Figure 1:**
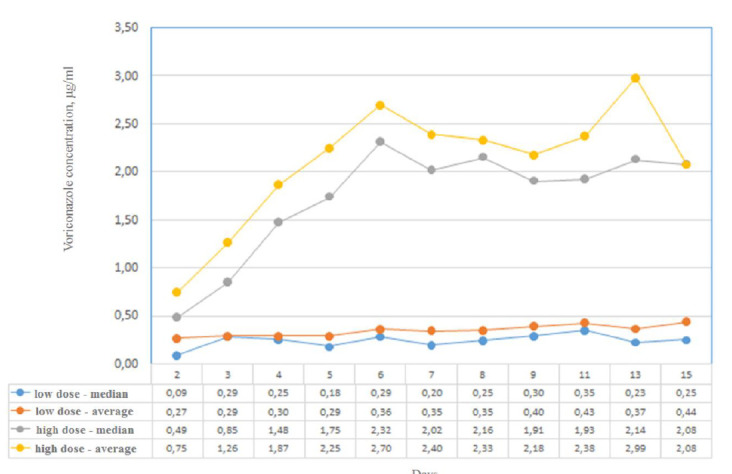
Medians and mean values of residual serum concentration of voriconazole by days from the start of prophylaxis in comparison groups

Average range of a neutropenia episode was 26 days (min:13 and max:51 days). The median of the onset of the first signs of IFI was 22 days from the start of prophylaxis (ranging from 15 to 33 days). The residual serum concentration during 15 days of prophylaxis was determined in all patients (Figure 1). 

As shown in Figure 1, in the high-dose group, the residual serum concentration reached a plateau after six days of prophylaxis. The median and mean values of serum VRCZ concentrations in the low-dose group did not exceed 0.5 μg/ml.

Concentration of VRCZ in blood serum in the high-dose group ranged from 0 to 14.56 μg/ml, with an average level of 2.11 μg/ml while 85% of patients had an average value of residual serum concentration in the range of 1-6 μg/ml. 

**Figure 2:**
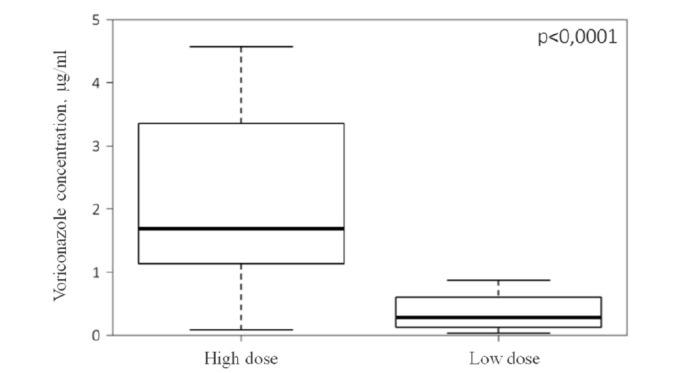
The dependence of the serum concentration of voriconazole on the dose of the drug

Concentration of VRCZ in blood serum of patients in the low-dose group ranged from 0 to 1.39 μg/ml with an average level of 0.35 μg/ml while they did not have an average value of residual serum concentration of 1-6 μg/ml (0%, *P*<0.0001) (Figure 2). 

**Figure 3:**
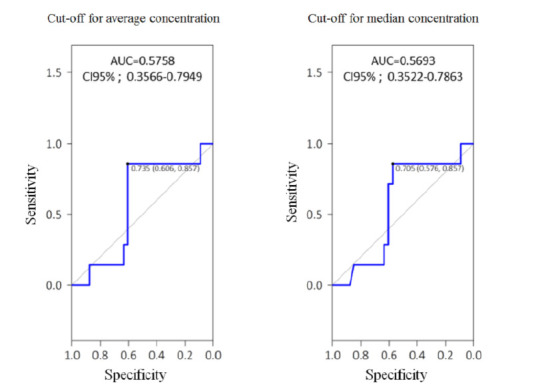
ROC analysis of the dependence of prophylaxis effectiveness on the concentration of voriconazole in serum

A ROC analysis was performed to determine the effective prophylactic concentration. The effective concentration was determined for average values ​​and median concentrations from observation points. For an average concentration, the diagnostic capacity was higher (Figure 3). The average concentration over the observation period at 11 measurement points was taken as an integral characteristic of the concentration of VRCZ in the serum of the patient since the AUC of the average concentration was greater than that of the median concentrations. According to the ROC analysis of effective concentration, cut-off values for prophylaxis of IFI was 0.74 μg/ml (with a sensitivity of 61% and a specificity of 86%). 

Likelihood of fungal infections is statistically significantly higher in patients with a residual serum concentration of < 0.74 μg/ml (*P*=0.0258). Concentration of < 0.74 μg/ml was observed in 19 episodes, in 6 of which (31.6%) patients became ill with IFI. A concentration of ≥ 0.74 μg/ml was observed in 21 episodes, of which only 1 (4.8%) fell ill with IFI. The risk of IFI development in patients with a residual serum concentration of VRCZ < 0.74 μg/ml was 6.63 times higher, compared to patients with a residual serum concentration of ≥ 0.74 μg/ml (*P*=0.0670) (relative risk =6.63, [RR] (95% CI)=0.88÷50.2). 

Study of *CYP2C19* gene polymorphisms revealed that five patients (in nine [ 22.5% ] episodes) had the genotype *1/*2 i.e. were intermediate metabolizers (*2 -intermediate enzyme activity), and 16 patients (in 31 [ 77.5% ] episodes) were fast or ultrafast metabolizers (*1-wild type-high enzyme activity or *17 [rs12248560]-ultra-high activity of the enzyme). In the study, there were no patients with a slow metabolism (*3 [rs4986893] characterized by reduced enzyme activity since it is very rare in the European population). Table 2 summarizes the analysis of the literature data on the study of *CYP2C19* gene polymorphism in children. 

**Table 2:** Study of polymorphisms of the CYP2C19 gene in children

Polymorphism	Type of polymorphism	Nucleotide substitution^1^	Amino acid substitution	Frequency in the population of 1000 genomes_Eur	Clinical relevance^2^	Reference
rs17885098	Synonymous	g.94762804C>T	p.Pro33=	С/С–0.002	Absent	PMC4051470
				C/T–0.139		PMC3310336
				T/T–0.859		
rs3758580	Synonymous	g.94842865C>T	p.Val330=	С/С–0.722	Absent	PMC4051470
				C/T–0.266		PMC3310336
				T/T–0.012		
rs3758581	Missense	g.94842866A>G	p.Ile331Val	A/A-0.002	Absent	PMC5625679
				A/G–0.133		PMC3310336
				G/G–0.865		
rs4244285	Synonymous	g.94781859G>A	p.Pro227=	G/G–0.722	Metabolism:	PharmGKB
				A/A–0.012	amitriptyline	PharmGKB
				A/G–0.266	citalopram	PharmGKB
					clomipramine	PharmGKB
					clopidrogrel	
rs17878459	Missense	g.94775165G>C	p.Glu92Asp	G/G-0.928	Absent	PMC6320343
				C/G–0.072		PMC4775436

1 Reference sequence: NC000010.11

2 According to data from ClinVar, a public database

Among the factors, the dose was associated with VRCZ metabolism (*P*<0.0001) and affected the residual serum concentration. Usage of cephalosporin antibiotics, in particular cefepime, was also associated with VRCZ metabolism (*P*=0.0415) which could be due to medication interactions.

In patients receiving high doses of VRCZ for prophylaxis, the residual serum concentration was significantly higher (*P*<0.0001) (Figure 2). Various factors, such as age (younger or older than 12 years, *P*=0.3714), gender (*P*=0.8278), *CYP2C19* polymorphism (presence of polymorphism rs4244285 [IM, *P*=0.5662], missense mutations in exons 2 or 7: polymorphism rs17878459 [*P*=0.3166] or rs3758581 [*P*=0.2414]), and the use of antibacterial medications (the most commonly used were analyzed) from the carbapenems group (in particular, meropenem, *P*=0.3852) or glycopeptides (vancomycin or teicoplanin, *P*=0.0905), or oxazoli-dinones (linezolid, *P*=0.9012) or colistin (*P*=0.5414), did not have a statistically significant effect on the level of residual serum concentration of VRCZ. Table 3 shows the distribution of factors in the two groups with different concentrations of the medication. 

**Table 3:** Distribution of factors in the two studied groups with different concentrations of voriconazole

Index	Concentration		*P*
	<0.74 (μg/ml)	≥0.74 (μg/ml)	
Total (n)	19	21	
Invasive fungal infections			
Invasive Fungal Infections	6 (31.6%)	1 (4.8%)	0.0258
Dose			
High dose	1	19	
Low dose	18	2	<0.0001
Polymorphism			
rs17878459-	17	20	0.4894
rs17878459+	2	1	
rs3758581-	4	7	0.3850
rs3758581+	15	14	
F/UM*	14	18	0.3421
IM** (rs4244285)	5	3	
Age			
<12 years	12	9	0.1991
≥12 years	7	12	
Gender			
Male	11	11	0.7263
Female	8	10	
Antibacterial medications			
FEP^1^-	15	9	0.0199
FEP+	4	12	
MEM^2^-	6	5	0.5826
MEM+	13	16	
VA^3^/TEC^4^-	11	8	0.2105
VA/TEC+	8	13	
LZD^5^-	18	20	0.9421
LZD+	1	1	
CL^6^-	17	17	0.4510
CL+	2	4	

* Fast/ultrafast metabolites, ** Intermediate metabolites, ^1^ Cefepime, ^2^ Meropenem, ^3^ Vancomycin, ^4^ Teicoplanin, ^5^ Linezolid, ^6^ Colistin

According to logistic regression, the dose appeared to be a highly significant factor influencing the residual serum concentration of VRCZ, *P*=0.0002 (odds ratio/OR=178.58, OR (95% CI)=19.14÷5945.54). Usage of cephalosporin had a low influence on the concentration of VRCZ, *P*=0.1992 (odds ratio/ OR=5.51, OR [ 95%CI ]=0.44÷136.10). 

There were no statistically significant differences between the two groups regarding the incidence of adverse events (*P*=0.0717); however, adverse events were more common in the high-dose group (Table 4). Adverse events, ranging from low to high increase in liver enzyme concentrations, were observed in three (15%) and 0 patients in the high-dose group and low-dose groups, respectively. An increase in enzymes of more than 5.1 times was registered as an adverse event (hepatic toxicity) of the third severity according to the common terminology criteria for adverse events [ 24 ]. 

**Table 4:** Adverse events in the two studied groups

Characteristic	High-dose group (n=20)		Low-dose group (n=20)		*P*
	n	%	n	%	
Adverse events (cases in total)	3	15	0	0	0.0717
Adverse events with I-II severity	2	10	0	0	0.1468
Adverse events with III-IV severity	1	5	0	0	0.3112

In the high-dose group, seven patients received intravenous VRCZ since they were not able to take the medication orally due to severe mucositis, while 13 patients took VRCZ tablets. There was a transition from the intravenous to the oral administration route due to the toxicity of one patient in the high-dose group. Moreover, for another patient, the reverse transition was made (from the oral to the intravenous route of administration). In the low-dose group, all patients took VRCZ tablets (n=20) except for two cases whose administration route was changed from oral to intravenous.

## Discussion

According to previous studies, the prophylactic use of VRCZ in children improves the survival of patients with AML and significantly reduces the number of IA [ 25 ]. To achieve VRCZ exposures comparable to those of adults, dosing in adolescents of 12-14 years old depends on their weight. Accordingly, they should be dosed like children if their weight is < 50 kg and dosed like adults if their weight is ≥ 50 kg; moreover, other adolescents should be dosed like adults [ 26 ]‎. In our study, we compared two different dosage regimens of VRCZ in children (including adolescents as described above) at high risk of IFI (mainly patients with AML and relapses of myeloid leukemia). Furthermore, we determined the residual serum concentration of VRCZ by high-performance liquid chromatography-mass spectrometry (HPLC-MS) and all patients underwent the determination of *CYP2C19* gene polymorphisms. 

Prophylaxis with VRCZ in low doses caused a higher number of breakthrough IFI (*P*=0.0375). Median of the onset of the first signs of IFI was 22 days from the start of prophylaxis, which confirms the leading role of the duration of neutropenia in the development of infectious complications. 

Among the many factors, to a large extent, the dose and, to a smaller extent, the use of antibacterial medications from the group of cephalosporins were directly associated with the metabolism of VRCZ, while the other factors did not have statistically significant clinical significance. However, this result could be due to the small number of studied patients. 

In the high-dose group, the residual serum concentration reached a plateau after six days of prophylaxis. According to the available published data, the level of residual serum concentration reaches a plateau after six days of therapy in most patients [ 27 ]. 

Ophthalmic disorders may not be sufficiently diagnosed due to difficulties in examining pediatric patients of a younger age group. Studies with an extended monitoring period are required to determine the long-term adverse effects of the use of VRCZ. 

In a study [ 19 ], a significant correlation was observed between oral doses and trough levels of VRCZ in patients of ≤ 6 years old (Spearman's rank correlation coefficient=0.4819, *P*=0.027). Patients of ≤ 6 years old needed a significantly higher median dose of PO VRCZ to maintain therapeutic trough levels, compared to older patient groups (8.9 vs. 4.2 mg/kg/dose, *P*<0.001). In our study, there were only 4 patients (in 5 episodes) aged ≤6 years. 

According to a previous research, the route of administration of the agent could affect the residual serum concentration [ 20 ]. In this study, out of the 33 patients who initially received VRCZ PO (13 and 20 in the high-dose and low-dose groups, respectively), 20 patients had a low concentration (1 and 19 in the high-dose and low-dose groups, *P*<0.001), and six of them fell ill with IFI (all in the low-dose group). Among the seven patients who initially received VRCZ IV, 0 patients had a low concentration (PO versus IV, *P*=0.0036) and none of them developed IFI. However, all seven patients who received VRCZ IV were in the high-dose group (i.e. received a high dose of VRCZ); therefore, the effect of the dose is noticeable and it is not possible to prove the effectiveness of routes of medication administration on the residual serum concentration. 

Based on the results of a previous study [ 20 ], VRCZ was used for prophylaxis at doses of 5 and 10 mg/kg/d orally, and it was found that it is better to use a higher dose. In this study, we used high doses of VRCZ and significantly different reults. However, the present study was conducted in our Center (only one center); therefore, we had a small number of patients for 2.5 years. In addition, some patients entered the study again (re-randomization) which could affect the results and limit our study. The homogeneity of the group, due to re-randomization, could affect the results of studying polymorphisms of the *CYP2C19* gene (the effect had no statistical significance on the residual serum concentration of VRCZ). An increase in the number of patients would increase the duration of the study which could make its conduction difficult. However, further research is needed to determine the optimal dosage regimen of VRCZ in children to prevent IFI. 

## Conclusion

Likelihood of fungal infections was statistically significantly lower in children who prophylactically received VRCZ in high doses (*P*=0.0375) with a residual serum concentration of ≥ 0.74 μg/ml (*P*=0.0258). The residual serum concentration of VRCZ reached a plateau by day sixth of the treatment. In children, the dosage was the only highly significant factor affecting the metabolism of the medication. 

## Authors’ contribution

S.L.K. designed the study on the poultry field and collected and analyzed the data. K.S.B. and A.V.H. wrote the manuscript. A.A.M. contributed to the manuscript writing of the CYP2C19 polymorphism section. O.I.B. performed statistical analysis. V.A.M. translated the manuscript into English and edited the final manuscript. O.V.A. participated in the study design and supervised the study.

## Financial disclosure

The authors received no external funding for this study.
